# Diverse Roles of Semaphorins on T Cell Activation, Differentiation, Migration, and Effector Functions

**DOI:** 10.3390/cells15121047

**Published:** 2026-06-07

**Authors:** Heqing Ma, Abdelilah S. Gounni, Ruey-Chyi Su, Sam K. P. Kung

**Affiliations:** 1 Department of Immunology, Rady Faculty of Health Sciences, Max Rady College of Medicine, University of Manitoba, Winnipeg, MB R3E 0W2, Canada; 2 National Sexually Transmitted Blood Borne Infection Laboratory Division, National Microbiology Laboratory, Public Health Agency of Canada, Winnipeg, MB R3E 3M4, Canada; ruey.c.su@phac-aspc.gc.ca

**Keywords:** semaphorins, T cells development, functions, immune regulations

## Abstract

**Highlights:**

**What are the main findings?**

- Multiple members of semaphorin family are involved in regulating diverse T cell responses.
- Dys-regulation of the semaphorin associated with multiple human diseases.

**What are the implications of the main findings?**

- Similar novel immune-regulatory functions of semaphorins may exist in natural killer cells.
- A good understanding of the semaphorin and its cognate receptor(s) signaling pathway will reveal novel therapeutic targets.

**Abstract:**

Semaphorins are a large family of proteins originally identified for their roles in axon guidance during neural development. Recent findings have established the importance of semaphorins members in modulating diverse immune responses of T cells in vitro and in vivo. Class 3 semaphorins, typified by Sema3A, signal through Neuropilin-1 and Plexin-A receptors in an activation-dependent manner, suppressing effector proliferation while promoting regulatory T cell stability and shaping cytokine profiles in autoimmunity and cancer. Sema3E and Sema3F similarly fine-tune host defense and inflammation by directing Th1/Th17 responses or restraining aberrant chemotaxis. Class 4 members, such as Sema4A and Sema4D, engage Plexin-B1, Plexin-D1, and CD72 to deliver both “forward” co-stimulatory and “reverse” signals: they amplify CD4^+^ and CD8^+^ effector functions, support T helper-B cell crosstalk, and influence tumor immunity via receptor shedding and bidirectional signaling. Finally, although less well defined, class 7 Sema7A operates indirectly—through APCs and Tregs—to regulate inflammatory recall responses and Th1/Th17 driven pathology. Together, these semaphorin-mediated pathways underscore a complex, context-dependent network that balances protective immunity against immunopathology, offering novel therapeutic targets in autoimmunity, infection, and cancer.

## 1. Introduction

Semaphorins are a large family of proteins originally identified for their roles in axon guidance during neural development. They constitute a large family of conserved proteins that are sub-grouped further by their structural and sequence similarities [1]. Recent findings have established the importance of semaphorins in modulating diverse immune responses in a number of immune cell types in vitro and in vivo. Of interest, subfamilies of semaphorins are now known to modulate many different aspects of T cell biology. This review will focus on three semaphorin subfamilies: Class 3, Class 4, and Class 7, and their diverse roles in T cell activation, differentiation, migration, and effector functions.

## 2. Class 3 Semaphorins: Activation Dependent Modulation of T Cell Responses

### 2.1. Semaphorin 3A

Semaphorin 3A (Sema3A) was first identified as a chemo-repulsive guidance cue in inducing collapse and retraction of dorsal root ganglion neuron growth cones via Neuropilin-1/Plexin-A receptor complexes [2]. In the immune system, Sema3A was prominently expressed by activated T cells and dendritic cells (DCs) [3]. Serum levels of Sema3A were significantly reduced in patients with asthma, Crohn’s disease (both in relapse and remission), ulcerative colitis (UC), multiple sclerosis (MS), and systemic sclerosis (SS). Sema3A levels are negatively correlated with the severity, prognosis, and duration of the illness [4,5,6,7,8]. In individuals suffering from familial Mediterranean fever, a genetic autoinflammatory condition, a decrease in serum levels of Sema3A was associated with a specific reduction in their expression in the patients’ T cells [9]. Furthermore, Plexin-A1, Nrp-1, and Plexin-A4 were all seen increased in RA, an indicator of activation state of T cells [10], suggesting the reducing level of Sema3A might de-regulate T cells to acquire a hyperresponsive phenotype.

Multiple studies have reported the specific immunosuppressing effect of Sema3A on Th17 responses. Studies in mouse models have shown that Sema3A treatment reduced IL-17, IFN-γ, IL-4, and autoimmune IgG2a levels, while increasing IL-10 production, indicating a shift toward an anti-inflammatory state6,10. Nrp1-deficient CD4^+^ T cells showed enhanced proliferation and preferential differentiation into Th17 cells [11]. In the experimental autoimmune uveitis (EAU) mouse model, an upregulation of miRNA that suppresses Sema3A expression was linked to increased IL-17 levels and disease exacerbation [12].

In patients with celiac disease, Sema3A expression on CD4^+^CD25^hi^ cells was negatively correlated with anti-TG and anti-DGP antibody level in the serum [13]. Moreover, blocking of Sema3A/Nrp-1 axis led to enhanced anti-tumor migration and cytotoxicity of CD8^+^ tumor infiltrating T cells [14,15]. Similarly, patients with allergic rhinitis or allergic conjunctivitis who had received Sema3A exhibited reduced levels of IL-4, IL-5, IL-13, IL-17, TNF-α, and IFN-γ, accompanied by an increase in IL-10, which helped maintain epithelial barrier integrity and alleviate allergic responses [16,17]. In addition, recombinant Sema3A treatment led to lower secretion of IFN-γ and IL-2, upon CD3/CD28 stimulation, reinforcing its suppressive effects on T cell activation [3,10,18]. Phenotypic analysis further demonstrated that Nrp-1^+^ CD4^+^ T cells treated with Sema3A express higher levels of Foxp3 and IL-10, suggesting a Treg-like functional shift [10]. Phenotypic screening of recombinant Sema3A treated PBMCs showed that Nrp-1^+^ CD4^+^ T cells were more biased towards the “Treg-like” state, with higher levels of Foxp3 and IL-10 expression [10]. Indeed, Sema3A promoted the stability of CD4^+^CD25^+^ Tregs in the presence of inflammatory stimuli through NF-kB signaling pathway [19], and a similar Treg-inducing effect has also been described in the asthma model [4]. Blocking Nrp-1 suppressed Treg stability and T cell suppressing functions in a mouse model of sepsis [20,21]. Furthermore, Nrp-1 expression was also negatively associated with ER stress in type 1 Treg (Tr1) cells [22], and targeting Nrp-1 by either recombinant Anti-Nrp-1 or Nrp-1 shRNA knockdown reduced Treg expression of Foxp3, CTLA-4, IL-10, and TGFb and impaired the immunosuppressive ability. Consistent with the immunosuppressive nature of Sema3A, co-culturing resting or activated T cells, under either homeostatic or inflammatory environment, with recombinant Sema3A inhibited their cell proliferation. Blocking either the Sema3A or Nrp-1 receptor with monoclonal antibodies was found to be effective in rescuing the impaired T cell proliferation [3,17,20,23].

Sema3A has a chemo-repulsive effect on mice T cell migration. Recombinant Sema3A decreased adhesion of developing human thymocytes to thymus epithelial cells (TECs) and increased trans well migration towards laminin [24]. CRMP2, downstream of Nrp-1 signaling pathway, was expressed in human T lymphocytes at both mRNA and protein levels, and the expression level was positively correlated with the migration rate of primary T cells [25]. Consistently, human thymocytes incubated with Sema3A showed a decreased expression of CXCR4, and impaired CXCL-12 driven “attractive” migration [26]; and such effect was mitigated in a chemically induced diabetic model, which leads to a significant reduction in Sema3A and Nrp-1 expression on T cells [27]. Co-culturing B6 murine thymocytes with recombinant Sema3A alone induced a significant morphological change and reduced adhesion to BSA and trans-well migration capacity [28]. Binding of Sema3A to Nrp-1 impaired T cell migration and killing function against target tumor cells, as observed in both mice and human tumor-infiltrating T cells [14,15]. Moreover, tumor derived Sema3A could also mildly inhibit tumor adhesion in an Nrp-1 independent manner [18]. Collectively, Sema3A functions as a detachment signal that facilitates T cell egress from resident tissues while impairing their ingress into specific tissue sites.

### 2.2. Semaphorin 3E

Semaphorin 3E (Sema3E), initially named M-SemaH in mice, belongs to the class III semaphorin family and is expressed by bronchial epithelial cells, fibroblasts, endothelial cells, dendritic cells, and macrophages [29]. Unlike other Class 3 semaphorins, Sema3E binds to Plexin D1 at high affinity, enabling it to signal independently of the co-receptor Nrp-1. Originally linked to embryonic development of lungs, neural tube, and skeletal tissues [30], Sema3E has since been implicated in various physiological and pathological contexts, including tumor progression and immune regulation [31]. Recently, multiple studies have demonstrated the importance of Sema3E in inflammation, infections, and autoimmune conditions, suggesting that Sema3E is not merely a guidance cue in neuronal development but a critical immunoregulatory molecule in multiple innate immune cell types.

While direct effects of Sema3E on T cell functions have not been fully elucidated, studies of Sema3E deficient or tissue specific Plexin D1 deficient mice revealed the importance of the Sema3E/Plexin D1 axis on the regulation of T cell differentiation via other immune cell types. In allergen induced asthma models, Sema3E expression is negatively correlated with disease severity [32], and selective deficiency of PlexinD1 in interstitial macrophages resulted in exacerbated disease features, including heightened airway hyperresponsiveness, increased eosinophil and leukocyte infiltration, elevated allergen-specific IgE, goblet cell hyperplasia, and amplified Th2/Th17 cytokine responses [33]. PlexinD1 deficient macrophages displayed reduced IL-10 expression, both at baseline and after HDM challenge, suggesting that impaired anti-inflammatory macrophage signaling underlies the worsened pathology [33]. In addition, Sema3E^−^/^−^ mice showed increased CD11b^+^ pulmonary DCs, which upon adoptive transfer, enhanced Th2 and Th17 responses in wild-type recipients [34]. Sema3E treatment modulated allergic cytokine production, reducing Th2-associated cytokines (IL-4, IL-5, IL-9) and IL-17A, while restoring a more balanced Th1/Th2 profile [35].

In Chlamydia muridarum lung infection, exogenous Sema3E enhanced bacterial clearance and reduced tissue pathology while promoting Th1 and Th17 responses (increased IFN-γ and IL-17) and reducing Treg frequencies. Sema3E deficiency resulted in impaired Th1/Th17 responses, enhanced Th2 skewing, increased Tregs, and dysregulated DC cytokine production (decreased IL-12, increased IL-10). Adoptive transfer of Sema3E sufficient DCs, but not Sema3E deficient DCs, restored protective Th1/Th17 immunity, indicating that Sema3E programmed DCs to promote effective T cell responses [36,37].

In contrast, during Leishmania major infection, Sema3E deficient mice exhibited enhanced resistance with stronger Th1 responses and reduced Treg and IL-10 levels. Although macrophage intrinsic parasite control was unchanged, Sema3E deficiency enhanced DC maturation and IL-12 production, improving their ability to drive Th1 differentiation [38].

PlexinD1 was highly expressed on double positive and recently selected CD69^+^ thymocytes, while Sema3E was present in both the cortex and medulla. Genetic deficiency of either Sema3E or PlexinD1 disrupted corticomedullary organization and altered thymocyte migration [39]. At the molecular level, Sema3E-PlexinD1 signaling inhibited activation of the small GTPase Rap1, a central regulator of integrin activation. Upon chemokine or TCR stimulation, Rap1 promoted high-affinity LFA-1 conformation and stabilized adhesion to ICAM-1 through RAPL and Mst1. Engagement of PlexinD1 suppresses Rap1 activation via its intracellular GAP domain, thereby limiting LFA-1 mediated adhesion and reducing thymocyte-stromal cell contacts. This modulation directly affected immunological synapse formation and lymphocyte migration [40].

### 2.3. Semaphorin 3F

Sema3F is constitutively expressed in human thymus, encompassing both developing T cells and various thymic compartments. Although the literature remains limited, direct evidence has been found on the involvement of Sema3F in T cell activity. In the human thymus, both Sema3F and its receptor component Nrp-2 were expressed in developing T cells as well as stromal compartments, and exogenous Sema3F inhibits CXCL12- and S1P-induced migration of human T cell precursors, which could be reversed by blocking Sema3F/Nrp-2 interaction [41]. These observations suggested that Sema3F regulated thymic positioning or egress by dampening responsiveness to key migratory cues. A study using human glioblastoma cell line U87MG showed that Sema3F suppressed Akt-mTOR signaling, disrupted mTORC1/2 complex formation, and induced cytoskeletal collapse, providing an indirect plausible explanation for its antimigratory effects [42].

Beyond precursor migration, the Sema3F/Nrp-2 axis might also contribute indirectly to immune regulation: Nrp-2 on dendritic cells modulated DC driven T cell activation [43], and Nrp-2-dependent suppressive activity of CD9^+^ Bregs has been linked to Sema3F expression on CD4^+^ T cells [44]. Taken together, current evidence supported Sema3F as a negative regulator of T cell behavior, particularly trafficking, while leaving unresolved the extent to which it directly controls mature T cell activation and effector differentiation.

## 3. Class 4 Semaphorins: Multifaceted Orchestrators of T Cell Activation, Differentiation, and Effector Function

### 3.1. Semaphorin 4A

In systemic lupus erythematosus and rheumatoid arthritis, CD4^+^ T cells exhibited increased Sema4A expression that correlated with disease severity [45]. Similarly, in an experimental autoimmune myocarditis model, mice lacking Sema4A were protected from heart inflammation, displaying a shift toward anti-inflammatory IL-4 and IL-10 production and failing to transfer disease via Sema4A-deficient CD4^+^ cells [46]. In multiple sclerosis plaques, the Sema4A expression co-localized with CD11b and CD4 [47], and elevated serum Sema4A aligns with stronger Th17 skewing and higher IL-17A, while Sema4A deficient animals showed milder EAE; restoring wild-type Th17 cells reinstates disease, underlining Sema4A’s critical role in driving pathogenic Th17 responses [48,49,50]. Likewise, Systemic Sclerosis (SS) patients demonstrated increased Sema4A that amplifies IL-17, IL-21, and IL-22 release by CD4^+^ cells [51]. In an arsenic-induced hepatotoxicity mouse model, blocking Sema4A mitigated the hepatotoxicity, accompanied by the down regulation of p-AKT2 or NF-κB p65, and NLRP3 inflammasomes [52]. Recombinant Sema4A acted as a potent co-stimulatory signal for CD4^+^ T cells: when co-cultured with T cell, it enhanced T cell proliferation and elevates secretion of key cytokines such as IL-17, IL-21, and IL-22—effects seen in both healthy donor cells and those from patients with autoimmune disease [50,51].

Plexin-B1, the primary Sema4A receptor, was constitutively expressed on human CD4^+^ T cells, whereas Sema4A itself was highly produced by dendritic cells (DCs) and co-localized with CD11b^+^ DCs and CD4^+^ T cells at the immunological synapse [48,50,53,54]. This close proximity during antigen presentation underscored Sema4A’s critical role in T cell priming. Besides, Sema4A expression could be induced in CD4^+^ T cells by anti-CD3/CD28 stimulation [51], and recombinant Sema4A enhanced CD4^+^ T cell proliferation in mixed lymphocyte reactions, demonstrating its potent co-stimulatory function [50,55,56]. Furthermore, Sema4A expression on T cells was upregulated upon activation and remained elevated throughout T helper cell differentiation [50,57], suggesting that Sema4A modulated CD4^+^ T cell responses beyond initial priming. Effects of Sema4A on other CD4^+^ subsets were highly dependent on the surrounding cytokine milieu and receptor engagement. For instance, under Th2-polarizing conditions (anti-IFNγ + IL-4), CRTH2^+^ memory Th2 cells upregulated Sema4A and required it to achieve robust IL-4 and IL-5 production. In this setting, blocking the Sema4A/Plexin-D1 axis diminished Th2 and Th17 cytokines, while paradoxically enhancing Th1 markers T-bet and IFNγ [53,56,57]. In contrast, other work showed that Th1-biased CD4^+^ cells expressed more Sema4A than their Th2 counterparts and that Sema4A-deficient Th1 cells exhibited reduced T-bet, IFNγ, and IL-12Rβ2—a finding that suggested Sema4A could reinforce Th1 differentiation in certain contexts [56]. Consistently, in OVA induced asthma, loss of Sema4A led to exaggerated Th2 (and even Th17) responses, whereas administration of exogenous Sema4A ameliorated airway inflammation and reduced IL-4, IL-5, IL-13, and IL-17 levels [54,58,59]. Taken together, these findings indicated that Sema4A could be a co-stimulatory molecule for CD4^+^ T cells, which, depending on specific environmental contexts, helped shape helper T cell polarization and effector functions according to receptor usage and surrounding cytokines.

CD8^+^ T cells expressed higher levels of Sema4A and its receptor Plexin-B1 than CD4^+^ subsets, and the expression of Sema4A was further increased upon activation with LM-OVA or anti-CD3/CD8 stimulation [60]. Although proximal TCR signaling remained intact in Sema4A deficient CD8^+^ cells, these cells exhibited profound functional impairments in IFN-γ and TNF-α production, production of cytotoxic effector molecules (granzyme B, perforin, and FasL), as well as the lineage defining transcription factors T-bet and Eomes [60]. Mechanistically, loss of Sema4A disrupted mTORC2 activation, leading to weaker pathogen-specific responses [60]. In tumor contexts, high Sema4A expression correlated with enrichment of T cell activation and differentiation gene signatures in LUAD and HNSCC, and administration of recombinant Sema4A enhanced CD8^+^ T cell infiltration into these tumors [61]. Clinically, patients with elevated Sema4A levels demonstrated better outcomes following anti–PD-1 therapy and reduced tumor burden in preclinical models, highlighting Sema4A as a promising target to boost CD8^+^ T cell-mediated cancer immunity [61].

Increasing number of studies have illustrated the importance of Sema4A on Treg cells. In Sema4A deficient mice, a decreased Treg/Teff ratio was reported, along with the heightened allergic airway response when challenged by OVA [58]. Administering recombinant Sema4A before ischemia reperfusion injury increased CD25^+^Foxp3^+^ Treg frequencies and IL-10 production in the kidney, which in turn, lowered TNF-α, IL-6, and CCL2 levels and protected the animals against tissue damage [62]. Similarly, exogeneous Sema4A increased CD25^+^Foxp3^+^ cell subsets in HuT102 cutaneous T lymphocyte cell line and human PBMC cultures [63], and Sema4A released by plasmacytoid dendritic cells (pDC) expanded Nrp1^+^ Treg cells during early life PVM infection in mice and prevented the host from viral bronchiolitis and asthma [55]. The detailed mechanism on how Sema4A interacted with Treg cells remained to be revealed, yet current research has found that Sema4A bound to the Nrp1 receptor to potentiate Treg function and survival by modulation of Akt-mTOR signaling [62,64], and Nrp1-deficient Tregs failed to suppress antitumor immune responses in mice [64].

### 3.2. Semaphorin 4D

Since Semaphorin 4D was first found on human T and B cells in the 1990s [65,66,67], it has been connected with immunity and intensively studied for 3 decades and has been well recognized as a key T cell regulator. Sema4D binds two principal receptors—Plexin-B1, which generally expressed across T cell subsets [61,63,68,69,70]; and CD72, which was present on a minor T cell subset but critical for TCR-mediated proliferation [69,71,72,73].

Notably, a naturally occurring K849T missense variant of Sema4D, identified in a family with primary sclerosing cholangitis, disrupted Sema4D-receptor interactions and impaired IFN-γ production upon T cell stimulation, underscoring the functional importance of these binding events [74]. Beyond classic “forward” signaling, membrane bound Sema4D itself could act as a receptor: crosslinking Sema4D on T cells enhanced Lck/ZAP-70 phosphorylation, amplifying antigen-receptor signaling cascades [75]. Concurrently, Sema4D underwent proteolytic shedding by MMP-2, MMP-9, and MMP-14, releasing a soluble ectodomain that retained biological activity—correlating with enhanced CD8^+^ cytotoxicity and cytokine secretion [72,76,77]. CD45 has also been implicated in Sema4D shedding on human T cells, where it promoted homotypic adhesion and may further regulate receptor availability [66]. Furthermore, Sema4D was involved in dendritic cell-T cell priming. In Sema4D^−/−^ mice, DC maturation and costimulatory molecule upregulation were blunted, resulting in diminished CD4^+^ and CD8^+^ T cell proliferation and cytokine output (e.g., IFN-γ, IL-4) following MOG or OVA challenge [78,79]. Together, these different signaling mechanisms positioned Sema4D as a multifaceted regulator of T cell activation, differentiation, and effector function.

Sema4D deficient mice showed lower infiltration of CD4^+^ and CD8^+^ T cells, coupled with reduced levels of inflammatory cytokines and chemokines like IL-1β, IL-6, IFN-γ, CXCL2, and CXCL5 at the hypersensitivity site in a contact-induced hypersensitivity model [80]. In vitro, Sema4D deficiency also impaired the recall T cell expansion from spleen monocytes under repeating OVA323–339 peptide challenge [81]. Furthermore, Sema4D-deficiet T cells exhibit decreased production of IL-12, IFN-γ, IL-2, and TNF-α, which in turn promoted the survival in graft-versus-host disease (GVHD) models [82]. Upon activation, the expression level of Sema4D on CD4^+^ T cells decreased, while it remained stable in other T cell subtypes [71], indicating a unique association between Sema4D and CD4 T cell activity. Indeed, recombinant human Sema4D have been shown to increase the population of CD3^+^CD4^+^CD25^hi^ T cells [69], and Sema4D^−/−^ mice displayed striking deficits across Th1, Th2, and Th17 lineages, including reduced IL-13, IL-5, IL-6, and IL-17 in bronchoalveolar lavage following OVA challenge [81,83]. Furthermore, splenocytes from Sema4D^−/−^ mice exhibited lower proliferation rates, reduced production of IL-4 and IL-10, a lower population of CD44^+^CD4^+^ T cells, and an increase in apoptotic CD4^+^ T cells [78], highlighting the importance of endogenous Sema4D in enhancing CD4^+^ T cell function and survival. Clinically, elevated soluble Sema4D levels in ankylosing spondylitis patients drive Th17 differentiation, further underscoring Sema4D’s pivotal role in sustaining CD4^+^ T cell function and survival [84].

Effective humoral immunity hinged on CD4^+^ T cell help, and Sema4D was critical in this partnership. In Sema4D^−^/^−^ mice challenged with horse apoferritin, serum IgG levels were markedly lower and glomerular injury was attenuated, reflecting defective B-cell activation and antibody production [78]. This impairment was further supported by observations of delayed B cell responses and reduced in vitro immunoglobulin production upon CD40 stimulation [85]. Likewise, Blocking Sema4D in naïve B activated T cell co-cultures similarly decreased plasmablast formation [86]. In co-culture experiments using naïve B and polyclonal activated T cells, blocking Sema4D also significantly decreased the number of plasmablasts [86]. Conversely, transgenic mice overexpressing Sema4D showed increased B cell proliferation and immunoglobulin production (IgM and IgG1) following IL-4 and anti-CD40 or LPS stimulation [87], and soluble Sema4D released from activated T cells was found to retain activities to enhance B-Cell response in MRL/lpr mice model [88]. Collectively, these studies demonstrated that Sema4D is vital for T cell-B cell interactions, influencing B cell activation, proliferation, and antibody production.

Sema4D is also crucial for the proper functioning of CD8^+^ T cells. For instance, Sema4D-deficient mice exhibited lower numbers of CD8^+^ T cells and higher numbers of regulatory T cells in the spleen [81]. These mice also showed impaired activation and differentiation of hapten-specific CD8^+^ T cells in response to oxazolone challenge [80]. Human patients with myocardial infraction showed an increased level of soluble Sema4D, and co-culturing with recombinant Sema4D increased cytokine production of CD8^+^ T cells from these patients [89]. Moreover, blockade of Sema4D with the monoclonal antibody fostered myeloid-derived suppressor cell mediated suppression, driving an overall immunosuppressive T cell phenotype [90]. In HIV-1 infection mice models, diminished Sema4D on CD8^+^ T cells correlated with immunosenescence, exhaustion, and higher viral loads, highlighting its role in sustaining CD8 T cell immune response [91].

Sema4D also played a critical role in regulating γδ T cell function, primarily through interactions with its receptor Plexin-B2 on neighboring tissue cells. In the skin, dendritic epidermal γδ T cells (DETCs) constitutively expressed CD100 and further upregulated it upon activation, enabling direct engagement with Plexin-B2 on keratinocytes and optimized γδ T cell activation through ERK and cofilin signaling pathways [92]. In the intestinal epithelium, the Sema4D expressing γδ T cell interacted with Plexin-B2 on epithelial cells and carried protective functions against DSS induced colitis in a mice model [93]. On the other hand, γδ T cell derived Sema4D contributed to pathology in certain contexts. In medication related osteonecrosis of the jaw (MRONJ), γδ T cells accumulated in lesions and expressed high levels of Sema4D, which promoted inflammatory cytokine production such as TNF-α. Neutralization of Sema4D or genetic absence of γδ T cells reduced disease severity, suggesting that soluble Sema4D drove pathogenic inflammation [94].

Across several viral infections, including HBV, HIV-1, HTNV, and HTLV-1, Sema4D (and its low-affinity receptor CD72) was upregulated on CD8^+^ T cells relative to healthy controls [77,95,96,97,98]. Functionally, CD72 blockade dampened anti-HBV CD8^+^ responses, whereas recombinant soluble Sema4D accelerates viral clearance and enhanced intrahepatic CD8^+^ activity in mice [77]. In hepatitis C virus infection, overexpression of Sema4D also correlated with increased IFN-γ and TNF-α expression in a CD72 dependent manner [99].

In the context of cancers, Sema4D expression on TILs often indicated a more exhausting state: Its upregulation in diverse cancers (e.g., liver, colon, breast, lung, head and neck) correlated with elevated checkpoint receptors (PD-1, TIGIT, TIM-3, LAG-3) on CD8^+^ T cells [100]. Knockdown of tumor derived Sema4D reduced CD8^+^ exhaustion, and anti-Sema4D antibodies synergized with PD-1 blockade to reject mouse oral squamous cell carcinoma (MOC1), and Lewis lung carcinoma (LLC), and non-small cell lung carcinoma in preclinical models [101,102].

Current evidence indicated that Sema4D regulated T cell migration predominantly in a context dependent and often indirect manner. In oral lichen planus, soluble Sema4D is elevated locally and systemically and promotes CD8^+^ T cell lesional trafficking by inducing CXCL9 and CXCL10 expression in oral keratinocytes through Plexin-B1 dependent Akt–NFκB signaling [72]. Consistent with a broader role in inflammatory cell accumulation, Sema4D deficiency attenuated contact hypersensitivity and reduced CD4^+^ and CD8^+^ infiltration at challenge sites [80]; however, these effects likely reflected multiple defects in adaptive priming, effector differentiation, and innate inflammatory amplification rather than a purely T cell intrinsic migratory defect.

In tumor microenvironments, Sema4D appeared to restrict immune cell penetration. Early in vitro studies suggested that soluble Sema4D could directly inhibit immune cell migration [103]. Later studies showed that antibody mediated neutralization of Sema4D enhanced monocyte and T cell infiltration into tumors [104], possibly due to the inhibition of Sema3D diminished the recruitment and suppressive function of polymorphonuclear myeloid derived suppressor cells [100]. Together, these findings supported a model in which Sema4D regulated tissue access of T cells, mainly by remodeling local inflammatory and stromal cues, with direct effects on T cell motility still incompletely defined.

## 4. Class 7 Semaphorins

### Semaphorins 7A

Semaphorin 7A (Sema7A) was highly expressed on human thymocytes, particularly within the CD3 dull population and most prominently on CD4^+^CD8^+^ double positive cells [105]. Although its immunomodulatory functions were less well defined than those of other semaphorins, several observations pointed to an indirect role in T-cell regulation. For example, Sema7A deficient T cells from immunized mice failed to mount inflammatory responses upon antigen rechallenge, despite showing normal proliferation [106]. In rheumatoid arthritis patients, elevated Sema7A levels in blood correlated with increased IFN-γ, IL-22, IL-17, and upregulation of RORγt and T-bet in CD4^+^ T cells, implicating Sema7A in Th1/Th17 driven pathology [107].

Unlike other semaphorins, Sema7A signaled through Plexin-C1 and β_1_ integrin on non-T cell lineages, yet common T cell subsets have not been shown to express these receptors. During T cell–macrophage synapse formation, Sema7A colocalized with GM1 glycosphingolipids and α_1_ integrin at the interface [106], suggesting a role in antigen presentation. Indeed, although Sema7A^−/−^ CD4^+^ T cells proliferated normally after DC or peptide stimulation, they secreted less IL-6 and TNF-α and induced weaker inflammatory responses in vivo [106]. Consistent with this indirect mechanism, exogenous Sema7A added directly to T cell cultures failed to alter proliferation or cytokine output [108]. Moreover, Sema7A^+^ regulatory T cells produced less IL-10, and transferring these cells into TGFβ1–treated Sema7A^−/−^ hosts precipitates lung fibrosis [109], indicating that the effects of Sema7A on immune regulation were mediated via antigen presenting cells and Tregs, rather than through direct T cell receptor engagement.

## 5. Perspectives and Future Directions

**Figure 1:**
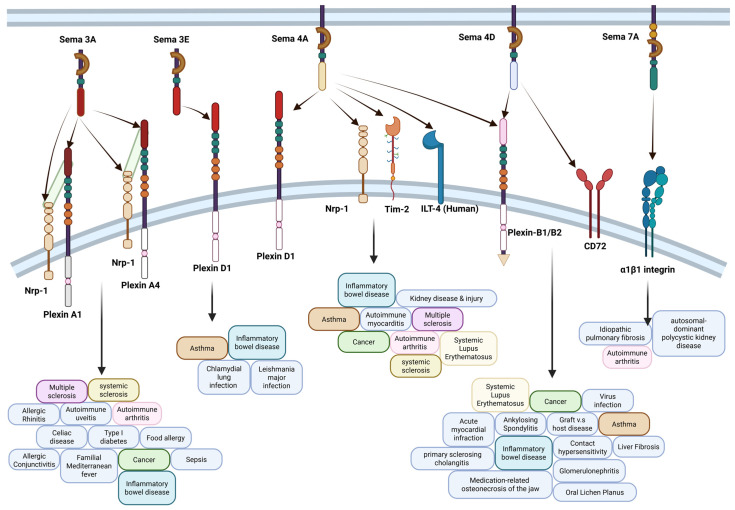
Disease associated with semaphorin-mediated regulation of T-cell responses. Schematic overview of human diseases and pathological conditions in which semaphorin–T cell interactions have been implicated. Diseases involves multiple semaphorins are labeled in different color. Semaphorin signaling pathways contribute to the regulation of T cell activation, differentiation, migration, immune synapse dynamics, and effector function across a broad spectrum of inflammatory, autoimmune, infectious, fibrotic, and malignant diseases. Dysregulated semaphorin signaling has been associated with allergic diseases, autoimmune and inflammatory disorders, fibrotic diseases, infectious diseases, graft-versus-host disease, and multiple forms of cancer.

The emerging roles of semaphorins in regulating T cell effector functions suggest considerable therapeutic potential across a broad range of immune-mediated diseases. Several semaphorin pathways appear particularly relevant in conditions characterized by dysregulated T cell subset balance, including autoimmune disorders, allergic inflammation, and chronic inflammatory diseases (Table 1, Table 2 and Table 3, Figure 1). For example, the immunosuppressive properties of Sema3A and the regulatory functions of Sema3E in asthma and colitis models (Table 1) suggest that enhancing class 3 semaphorin signaling may represent a promising strategy for limiting excessive inflammatory responses while preserving immune homeostasis. In contrast, targeting co-stimulatory semaphorins such as Sema4A and Sema4D (Table 2) may provide opportunities to modulate pathogenic T cell activation or improve antitumor immunity depending on disease context. Importantly, many clinical and pre-clinical studies targeting semaphorin signaling, or adopting exogenous semaphorins as a treatment method has shown effectiveness, highlighting the potential of semaphorin signaling pathways as a therapeutical method.

**Figure 2:**
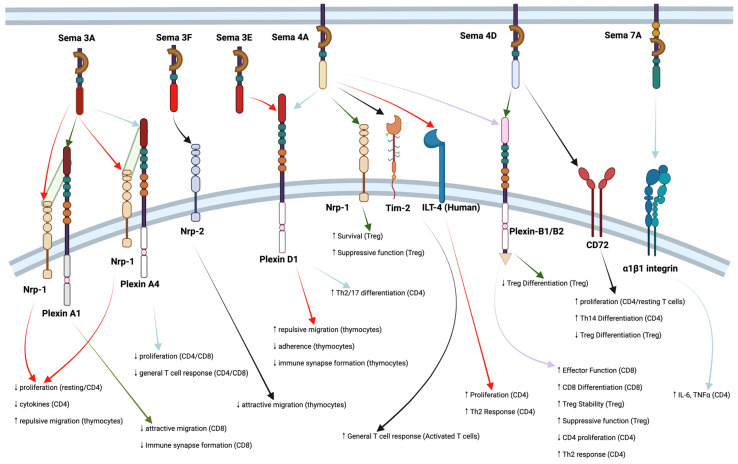
Semaphorin signaling axis regulating T cell biology. Semaphorin ligands, their cognate receptors expressed on T cell subsets, and the major T cell functions regulated by the specific interactions are connected by arrows with the same color. Class 3 semaphorins, including Sema3A, Sema3E, and Sema3F, primarily signal through neuropilin-1 and -2 and plexinA1, A4, and D1 to regulate thymocyte migration, integrin mediated adhesion, immune synapse formation, and T cell activation. Sema3E–PlexinD1 signaling additionally modulates Rap1/LFA-1 associated adhesive pathways involved in thymocyte positioning and T cell–APC interactions. Class 4 semaphorins, particularly Sema4A and Sema4D, interact with multiple receptors including Plexin-B1/B2, Plexin-D1, Tim-2, ILT-4, Nrp-1, and CD72 to regulate CD4^+^ and CD8^+^ T cell activation, proliferation, differentiation, effector function, and Treg stability. Sema7A signaling through α1β1 integrin contributes to inflammatory cytokine production and T cell-mediated immune responses.

**Figure 3:**
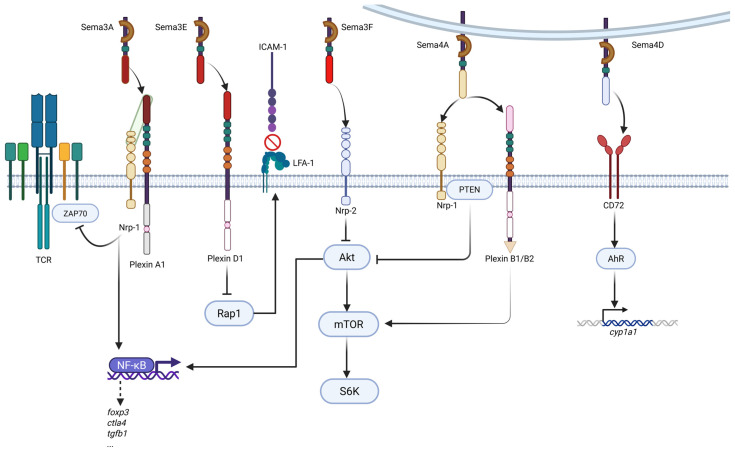
Major intracellular signaling pathways downstream of semaphorin receptors in T cells and thymocytes. Schematic overview of representative intracellular signaling pathways regulated by semaphorin–receptor interactions in T cells and thymocytes. Sema3A signaling through the Nrp-1/Plexin-A1 receptor complex inhibits activation of TCR associated ZAP70 protein and contributes to NF-κB-associated regulatory programs linked to Treg-associated gene expression, including *Foxp3, Ctla4*, and *Tgfb1*. Sema3E–PlexinD1 signaling suppresses Rap1 activation, thereby inhibiting LFA-1 mediated adhesion to ICAM-1 and regulating thymocyte migration and immune synapse dynamics. Sema3F signaling through Nrp-2 has been associated with suppression of Akt/mTOR/S6K signaling and inhibition of migratory responses. Sema4A exerts receptor-dependent effects, including PTEN-mediated suppression of Akt signaling downstream of Nrp-1 and activation of mTOR-associated pathways through Plexin-B1/B2 signaling. Sema4D signaling through CD72 has been linked to AhR-associated transcriptional responses, including induction of *Cyp1a1* expression and regulation of Th17/Treg balance.

However, current studies on semaphorins and T cells have focused mostly on observing phenotypic changes following the deletion or deficiency of semaphorins or their receptors. Although these studies have offered invaluable insights into novel roles of semaphorins in T cell regulations, they often were complicated by multiple (co)-receptors that some of these semaphoring members bind to, and thus, may be ambiguous for translational study trying to target specific pathway for therapeutic applications. For instance, Sema4A bound with Plexin-D1, Plexin-B2, TIM-2, Nrp-1, and a newly found receptor ILT-4 [53] (Figure 2). The Sema4A/Plexin-D1 axis activated mTOR pathways (Figure 3), and facilitated Th2/Th17 differentiation [57]; Sema4A/Plexin-B2 axis conferred partial immune regulatory functions by supporting Treg survival [63] and optimizing CD8 T cell activation at the same time [60]; whereas Sema4A/Nrp-1 axis was almost solely linked with Treg and immune regulation [62,64,110]. Such unique tuning mediated by different receptors might be an outcome of spatial and temporal expression of these receptors. To support this, a recent study showed that Plexin-B2 was expressed steadily at resting state, but its expression was gradually decreased after TCR activation; also, Nrp-1 was expressed exclusively after TCR stimulation [111] on human T cells, suggesting a possible explanation of the highly context-dependent effect of Sema4A on specific T cell responses.

Thus, it is crucial not only to understand the role of semaphorins in T cell activity but also to elucidate the specific signaling pathways that drive the observed alterations and the contextual cues that regulate receptor expression. These factors collectively could fine-tune and integrate multiple signals that would determine the final immune outcomes. Delineating the precise molecular mechanisms behind these dynamics under specific settings may provide new therapeutic opportunities to selectively modulate immune responses. For examples, targeting the Sema4A/Nrp-1 axis might enhance Treg function in autoimmune disorders, whereas inhibiting the Sema4A/Plexin-D1 axis may suppress pathogenic Th17 responses in inflammatory diseases.

In addition, some semaphorin family members undergo post-translational cleavages to generate distinct isoforms that exert different biological functions. For example, Sema3E is initially produced as a full-length 87 kDa protein and can be cleaved by furin or furin-like convertases at approximately a-a 560 [112,113]. This processing generates a 61 kDa isoform, p61-Sema3E, which exerts functions distinct from those of the full-length protein. In the nervous system, the full length Sema3E is found to inhibit choroidal neovascularization, which is the major cause of blindness in exudative age-related macular degeneration (AMD) [114]. Notably, p61-Sema3E has been shown to promote tumor growth and lung metastasis in mouse adenocarcinoma through activation of ERK1/2 signaling [113]. A furin-resistant mutant form of the 87 kDa full-length Sema3E suppressed tumor growth and angiogenesis [115,116]. Besides Sema3E, other Class 3 members may also be potential target for furin cleavage. Sema3F, for example, contains a conserved furin binding site at its C-terminal, and its processing has been shown to regulate its binding to the Nrp receptor [117]. Sema3A, 3B, and 3C could also been cleaved by furin [112,118,119], although its biological significance is still largely unknown. Moreover, Sema4D could also be cleaved, facilitated by multiple factors including gelatinases MMP-2, 9, and 14; membrane-tethered collagenase MT1-MMP; or metalloprotease ADAM17 [72,76,77,120,121]. The subsequent soluble forms of Sema4D are found to regulate various cell functions including angiogenesis, platelet collagen responses, and T cell functions. Further studies are needed to dissect how the expression of the semaphorin receptors and specific semaphorin isoforms induce downstream signaling pathways to regulate different immune cell functions. Such efforts will be essential for fully defining the therapeutic potential of semaphorin-based interventions.

T cell development and maturation seem to be another underappreciated aspect of semaphoring functions. Sema3E has distinct expression in both the cortex and medulla of the thymus, areas critical for T cell development and maturation, and the high expression of its receptor, Plexin D1, on DP and CD69^+^CD4^+^ single positive (SP) T cells further implicated its significance in thymic T cell functional maturation [34,35]. Similarly, Sema4A, together with Sema4D and Sema7A, was also expressed at varying levels in murine thymus. Sema4A protein was predominantly found in the thymus medulla and at the cortico-medullary boundary [122], regions critical for T cell development. During early T cell maturation, CD117^−^ DN1 cells, which represented a more multipotent T cell progenitor state, were largely positive for Sema4A. However, as these thymocytes progress through the double negative (DN) stages of maturation, the expression of Sema4A gene and protein gradually decreased [122]. Moreover, an increase in Nrp-1 expression during thymocyte maturation (TN3 to TN4) down-regulates CD25 [122]. These patterns, along with the fact that Sema4A reduction was a potential marker of thymocyte maturation, all suggested that Sema3E and 4A might be involved in the early stages of T cell progenitor differentiation and T cell education. Yet, to date, little is known about its exact role underlying T cell development and maturation. Investigating their potential roles in mediating interactions between thymic stromal cells and developing thymocytes, as well as the signaling pathways activated by their respective receptors, could provide valuable insights into their functions. Furthermore, understanding how semaphorin expression is regulated during thymocyte maturation and the downstream effects of their signaling could help elucidate their contribution to shaping a functional and self-tolerant T cell repertoire.

Semaphorin signaling has also emerged as an important regulator of tumor immunity, although its effects vary substantially across semaphorin family members and tumor contexts. Sema3A generally acts as an immunosuppressive factor, with its shown interfere with T cell migration, immune synapse formation, cytokine production, and cytotoxicity (Figure 2). The suppressive role in tumor microenvironment is further supported by the enhanced antitumor T cell responses after blocking the Sema3A/Nrp-1 axis [14,15]. In contrast, Sema4A has been associated with improved antitumor immunity in several settings, where Sema4A enhances CD8^+^ T cell infiltration, proliferation, cytotoxic activity, and responsiveness to anti–PD-1 therapy (Figure 2). Sema4D appears more context-dependent: while soluble Sema4D can support CD8^+^ T cell effector activity in some inflammatory or infectious settings [72,76,77,91], elevated Sema4D in tumors is frequently associated with immune cell exclusion [104], checkpoint receptor expression, and CD8^+^ T cell exhaustion [101,102]. These findings suggest that semaphorin pathways may influence both resistance and responsiveness to cancer immunotherapy by regulating T cell trafficking, effector function, exhaustion, and Treg-mediated suppression.

Despite the remarkable difference in the receptor systems T cells and natural killer (NK) cells utilize in their target recognitions, these two cell types share a lot of signaling and functional similarities. Currently, there are limited number of studies that have implicated immune-regulatory function(s) of semaphorins on NK cell biology (Table 4). First, NK cell transcriptional landscapes in endometrium showed that aberrant Sema3B activation coincided with reduced NK-cell abundance in the thin endometrium [123]. Second, Class 3 semaphorin Sema3E, produced by immature dendritic cells, acted as negative guidance cues that restrain activated NK-cell migration [124], while Sema3A indirectly enhanced NK recruitment and antitumor activity by reprogramming tumor associated macrophages toward an M1 phenotype [125]. Third, several class IV semaphorin pathways emerged as direct modulators of NK effector function: Sema4D enhanced NK–target cell adhesion, cytotoxicity, and IFN-γ production through interactions with CD72 or Plexin-B1/B2, and type I interferon amplified this axis in antiviral immunity [126,127,128]. Interestingly, tumor cells could also exploit Sema4D/PLXNB1 signaling on infiltrating myeloid and T/NK cells. In colorectal cancer, a metastasis-associated epithelial subset with elevated Sema4D showed increased Plexin-B1–Sema4D interactions with immune cells in liver metastases, and high Sema4D expression correlated with poor overall survival [129]. In contrast, PlexinB1 expression on glioma cells appeared to facilitate NK mediated killing [130], highlighting context dependent outcomes of the same ligand–receptor pair. Fourth, beyond membrane-anchored semaphorins, soluble Sema5A functioned as a potent activator of NK proliferation and pro-inflammatory cytokine production, contributing to systemic immune activation in autoimmunity [131]. Finally, Sema7A was strongly induced during cytokine induced memory-like NK cell priming and supported the acquisition of heightened recall responses [132]. It will be of great interest to explore in greater details how specific Semaphorin members regulate NK cell development and/or effector functions. We would speculate that emerging parallels of some of these immune-regulatory functions of semaphorins on T cells may also exist in NK cells. A good understanding of how members of the semaphorin family regulate T and NK cell biology would reveal novel signaling pathways and therapeutic targets to manipulate specific T and/or NK cell responses in vivo.

## 6. Conclusions

Development, differentiation and effector functions specific T cell subsets are tightly regulated because of the critical importance of mounting a “correct” specific T cell function(s) in adaptive immunity. Members of the semaphorins family are emerging as critical regulators of T cell biology. In this review, we summarize and critically discuss recent discoveries regarding the regulatory and signaling roles of semaphorins in T cells. By integrating findings from current published work in this field, we provide a comprehensive overview of class III, IV, and VII semaphorins and their interactions with T cells. Specifically, we discuss how these semaphorins regulate T cell fitness, activation, differentiation, migration, effector functions, and interactions with other immune cells. Members of this family exhibit diverse and context-dependent roles in T cell mediated immunity, and have implications for autoimmune diseases, infections, and cancers (Table 1, Table 2 and Table 3). Besides the major semaphorins we have presented above, there are other understudied family members that need to be further explored. For instance, Sema5A was found to induce T cell proliferation and functional factor expression [131], and co-expressed with IL-17 A in biopsies of Chronic Spontaneous Urticaria [133]. Sema6B and 6D were also found associated with T cell activation and infiltration [134,135]. There are currently very limited number of studies that have examined the immune-regulatory function(s) of semaphorins on NK cell biology (Table 4). Because of the similarities of T cells and NK cells in some of their signaling pathways and effector functions, we speculate further that emerging parallels of some of these immune-regulatory functions of semaphorins on T cells may also exist in NK cells. A good understanding of how members of the semaphorin family regulate T and NK cell biology would reveal novel signaling pathways and therapeutic targets to manipulate specific T and/or NK cell responses in vivo.

**Table 1:** Involvements of Class 3 semaphorin-T cell interactions in diseases.

Semaphorin	Disease/Condition	Expression Change in Disease	Assessed Source	T Cell Alteration in the Disease	T Cell Related Effect of Sema Treatment	Evidence Type	Reference(s)
Sema3A	Allergic conjunctivitis	N/A	N/A	↑ Th1/Th2 responses	↓ T cell proliferation, ↓ IL-4, IL-5, IL-13, IL-17, TNFα, IFN γ, ↑ IL-10 by Sema3A treatment	Mouse	[16,136]
	Allergic Rhinitis	↓	Serum	↑ Th2 responses	↓ T cell proliferation, ↓ Th17 response, ↑ Treg	Human + mouse	[6,17,137]
	Autoimmune arthritis	↓	T cell/PBMC	↑ Nrp-1, Plexin-A1/A4 on CD4^+^ and CD8^+^ T cells	↓ IFN γ, IL-17 ↑ IL-10	Human + mouse	[10]
	Autoimmune uveitis	↓	Serum	↑ IL-17, TNF-α, and IL-1β	↓ Th17 by inhibitor of Sema3A-targeting miRNA	Mouse	[12]
	Cancer (COS-7, NY-ESO-1 tumor antigen)	Negatively correlates with T cell function	COS-7 Cell line	↓ cytokine production and cytotoxicity, ↓ CD8 T cell adhesion and immune synapse formation	↑ proliferation, cytokine production and target recognition	Human	[14,18]
	Celiac disease	↑	Serum/T cell	↓ Treg expressed Sema3A, ↓ Serum IgA	N/A	Human	[13]
	Diabetes	↓	T cells	↓ Total T cell count, ↓ DN thymocytes, ↑ CD4/CD8 SP T cells, ↓ Sema3A induced migration	N/A	Mouse	[27]
	Familial Mediterranean fever	↓	Serum/Treg	↓ Treg percentage	N/A	Human	[9,138]
	Food allergy	N/A	N/A	↑ ER stress in type 1 Treg (Tr1)	↓ ER stress, ↑ regulatory ability of Tr1	Human	[22]
	Inflammatory Bowel Disease	↓	Serum/Treg	↑ CD4 T cell frequency in intestinal tissue	N/A	Human	[5,139]
	Multiple sclerosis	↓	Serum/Treg	↑ Th1, Th17, and CD8^+^ T response	↑ T cell migration by overexpression of Sema3A signal transducer protein CRMP2	Human	[7,8,25,140]
		↑ Sema3A signal Transducing protein CRMP2	T cells				
	Sepsis	↑	Serum	↑ T cell anergy, ↑ Sema3A expression by T cells, ↑ Foxp3 expression in CD4T cells	↓ T cell anergy, ↓ Treg regulatory functions by blocking Sema3A/Nrp-1 Axis	Mouse	[20,21]
	Systemic sclerosis	↓	Serum/Treg	↑ Th2, Th17; ↓ Treg frequency	N/A	Human	[8,141]
Sema3C	Rheumatoid arthritis	↑	Synovial tissue/macrophage/synovial fibroblasts	↑ Th1, Th17, Tph; ↓ Treg frequency	N/A	Human	[10,136,142,143]
Sema3E	Asthma	↓	Airway DCs/macrophages	↑ Th2/Th17 response	↓ Allergic cytokine and antibody responses, ↓ DC priming of co-cultured T cells	Mouse	[32,33,34,138]
	IBD	↓ colonic tissue	Serum	↑ CD4 T cell frequency in intestinal tissue	↓ IL-4/10 ↑ IL-17/IFN γ	Human + Mouse	[139,144]
	Chlamydial lung infection	↑	Lung tissue	↑ Th1 Response under Sema3E Deficiency	↓ Treg, ↓ IL-2, IL-10; ↑ IFN γ	Mouse	[36,37]
	*Leishmania major* Infection	↑	Cutaneous site of infection	↑ CD4^+^ T cell response	↓ Th1 differentiation	Mouse	[38]

**Table 2:** Involvements of Class 4 semaphorin-T cell interactions in diseases.

Semaphorin	Disease/Condition	Expression Change in Disease	Assessed Source	T Cell Alteration in the Disease	Effect of Sema Treatment	Evidence Type	Reference
Sema4A	Asthma	↑	Lung tissue/DC/CD4 T cells	↑ allergic airway response, ↑ Th2 response, ↓ Tregs, in Sema4A deficient mice	↓ Th2 response, ↑ Tregs	Mouse	[54,55,58,59,140,145,146]
	autoimmune myocarditis	N/A	N/A	↑ IL-4 and IL-10 under Sema4A Deficient mice	N/A	Mouse	[46]
	Cancer (LUAD, HNSCC)	Positively correlates with survival	Tumor/T cells	↑ CD8 activation/infiltration by overexpression of Sema4A; Treg mediated tumor tolorange by blocking Sema4A/Nrp-1 Axis	N/A	Human + mouse	[61,64]
	IBD	↓	Serum	↑ CD4 T cell frequency in intestinal tissue	N/A	Human	[5,139]
	kidney ischemia reperfusion injury	N/A	N/A	↑ Th1, Th17, and γδ T cell responses	↓ TNFα, IL-6 and CCL-2, ↓ Akt-mTOR pathway, ↑ Treg	Mouse	[62,147]
	Multiple sclerosis	↑	Serum/DC/T cell	↑ Th17 response ↓ Th2 response	N/A	Human + mouse	[7,47,48,49,50,140]
	Rheumatoid arthritis	↑	Serum/CD4^+^ T cells	↑ Th1, Th17, Tph; ↓ Treg frequency	N/A	Human	[45,143]
	Systemic Lupus Erythematosus	↑	Serum/CD4^+^ T cells	↑ CD4^+^ T cell activation and T cell help	N/A	Human	[45,148]
	Systemic sclerosis	↑	Serum/Monocytes/CD4^+^ T cells	↑ Th17 response	N/A	Human	[51]
Sema4D	Acute myocardial infarction	↑	serum	↑ CD4^+^ T cell infiltration, Th1 response	↑ CD8 T cell effector functions	Human	[89,149]
	Ankylosing Spondylitis	Positively correlates with severity	serum	↑ IL-17, ↑ C-reactive protein	↑ CD4 T cell proliferation, ↑ Th17, ↓ Treg	Human	[84]
	Asthma	N/A	N/A	↑ Th2/Th17 response	↓ IL-13, IL-5, IL-6, IL-17a and TGF	Mouse	[32,33,34,81,138,146]
	Colitis	N/A	Showed expression on γδ T cells	↑ CD4 T cell frequency in intestinal tissue	↓ proliferation and γδT cells in Sema4D deficient mice	Mouse	[93,139]
	Contact hypersensitivity	N/A	N/A	↑ Hapten specific T cells	↓ CD4/CD8 T cell infiltration ↓ IL-1β, IL-6, IFNγ in Sema4D defecient mice	Mouse	[80,150]
	Glomerulonephritis	N/A	N/A	↑ Th1, Th17 responses	↓ IL-4/IL-10, ↓ CD4^+^ T cell ↓ T cell activation in Sema4D deficiency	Mouse	[69,151]
	Graft vs. Host Disease	Deficiency improves survival	T cells	↑ Th1, Th17 responses	↓ IL-2, IL-12, TNFα, IFNγ, ↓ T cell expansion under Sema4D deficiency	Mouse	[82,152]
	liver fibrosis	↑	liver tissue	↑ Th17, ↓ Tregs	↓ Th1, Th2, and Th17 populations, ↑ Tbet expression in Tregs in Sema4D deficient mice	Mouse	[83,153]
	Medication-related osteonecrosis of the jaw	N/A	N/A	↑ Infiltration of γδT cells	↓ TNFα in Sema4D deficient mice	Mouse	[94]
	Oral Lichen Planus	↑	OLP tissue/serum	↑ CD8^+^ T cell infiltration, ↑ Th17/Treg ratio	↑ CD8 T cell migration and infiltration	Human	[72,148,149,154,155]
	primary sclerosing cholangitis	K849T mutation of Sema4D	Autosom	↑ T cell proliferation ↓ IFNγ production	N/A	Human	[74]
	Systemic Lupus Erythematosus	N/A	Serum/T cells/B cells	↑ CD4^+^ T cell activation and T cell help	N/A	Mouse	[88,148]
	Cancer (multiple cancer types)	↑ Membrane bound isoform	Tumor/T cells/Bcells	T cell response varies upon cancer types	↑ CD8 T cell effector functions	Human	[100]
	Virus Infection						
	HBV	↑ on cells, ↓ in soluble form	CD4, CD8 T cells/NK cells	↓ CD8^+^ T cell	↑ T cell response and viral clearance	Human + Mouse	[77,156]
	Hepatitis C	↓	CD8 T cells	↓ CD8 T cells, ↓ effector/central memory ratio of CD8 T cells	N/A	Human	[99]
	HIV	Altered Differently in T cell subsets	T cells	Altered T cell activation, ↑ PD-1/PD-L1 on T cells	N/A	Human	[91,95,97]
	Human Hantaan Virus	↑	CD8 T cells	↑ CD8lowCD100^+^ T. ↓ CD8low CD100^−^ T cells	N/A	Human	[98]
	Lymphotropic Virus Type 1	↑	CD4 T cells	Altered CD4^+^ T cell clonal expansion	N/A	Human	[96,157]

**Table 3:** Involvements of Class 5 and 7 semaphorin-T cell interactions in diseases.

Semaphorin	Disease/Condition	Expression Change in Disease	Assessed Source	T Cell Alteration in the Disease	Effect of Sema Treatment	Evidence Type	Reference
Sema5A	Chronic Spontaneous Urticaria	↑	lesional skin tissue	↑ IL-17a	↑ IL-17a in healthy CD4 T cells treated by Sema5A	Human	[133]
	Rheumatoid arthritis	↑	Serum/T cells/NK cells	↑ Th1, Th17, Tph; ↓ Treg frequency	↑ T cell proliferation, cytokine production, ↑ RORgt and T-bet in T cells	Human	[131,143]
Sema7A	autosomal-dominant polycystic kidney disease	↑	Serum/ CD4 T cells	↑ Renal CD8^+^ and CD4^+^ T cells	N/A	Human	[158,159]
	Idiopathic pulmonary fibrosis	↑	Serum/CD4^+^ cells/CD19^+^ cells	↑ Th2, Th17, Treg responses	↑ IFNγ, IL-4, IL-17a, ↓ IL-10	Human, Mouse	[109,160]
	Rheumatoid arthritis	Positively correlated with severity	Serum/CD4 T cells	↑ IFNγ, IL-22, and IL-17	↑ RORgt and T-bet on CD4 T cells	Mouse	[107]

**Table 4:** Semaphorins in T and NK cells.

Common Cellular Functions Between T&NK Cells	T Cells	NK Cells
Pro-inflammatory cytokines (e.g., IFN-γ, TNF-α, IL-2, IL-7, etc.)	Sema3A↓ (ref: [3,6,10,16,17,18])	Sema3A↑ (ref: [125])
	Sema3E↑ (ref: [36,37]) ↓ (ref: [33,34,35])	?
	Sema4A↑ (ref: [48,49,50,51,53,56,57,60]) ↓ (ref: [54,58,59])	?
	Sema4D↑ (ref: [72,74,76,77,80,81,82,83,89])	Sema4D↑ (ref: [126,128])
	Sema7A↑ (ref: [106,107])	Sema7A↑ (ref: [132])
Anti-inflammatory cytokines (e.g., IL-10, TGFβ, etc.)	Sema3A↑ (ref: [6,10,19,20,21,22])	?
	Sema3E↑ (ref: [38]) ↓ (ref: [36,37])	?
	Sema4A↑ (ref: [55,62,63,64]) ↓ (ref: [46])	?
	Sema4D↑ (ref: [78]) ↓ (ref: [81,90])	?
	Sema7A↓ (ref:109])	?
Cell Differentiation	Sema3A↓ (ref: [11])	?
	Sema3E↑ (ref: [36,37]) ↓ (ref: [38])	?
	Sema4A↑ (ref: [48,49,50,56,57])	?
	Sema4D↑ (ref: [69,71,80,81])	?
Tumor infiltration	Sema3A↓ (ref: [14,15])	Sema3A↑ (ref: [125])
	Sema4A↑ (ref: [61])	?
	Sema4D↓ (ref: [104])	?
Cytotoxicity	Sema3A↓ (ref: [14,15])	?
	Sema4D↑ (ref: [72,76,77])	Sema4D↑ (ref: [126,130])
Migration	Sema3A↑ (ref: [24,25]) ↓ (ref: [26,27,28])	?
	Sema3E↑ (thymocytes) (ref: [39])	Sema3E↓ (ref: [124])
	Sema3F↓ (ref: [41])	?
	Sema4D↑ (ref: [72,80]) ↓ (ref: [100,103])	?
Proliferation	Sema3A↓ (ref: [11])	?
	Sema4A↑ (ref: [50,51,55,56])	?
	Sema4D↑ (ref: [78,79])	?
	Sema5A↑ (ref: [131])	Sema5A↑ (ref: [131])
Antigen presenting cell crosstalk	Sema3E↓ (ref: [40])	?
	Sema3F↓ (ref: [43])	?
	Sema4A↑ (ref: [47])	?
	Sema4D↑ (ref: [78,79])	?
	Sema7A↑ (ref: [106])	?
Exhaustion	Sema4D↑ (ref: [100])	?

↑/↓: Corresponding T cell functions up or down regulated by the indicated semaphorin. Conflicting observations may result from differences in disease context, experimental models, species, or tissue-specific microenvironments. ?: The involvement of corresponding semaphorin in mentioned aspect of NK biology is not clear.

## Data Availability

The information presented here were derived from publications available in the Pubmed domain.
